# Cerebellar Dysfunction in Adults with Prader Willi Syndrome

**DOI:** 10.3390/jcm10153320

**Published:** 2021-07-28

**Authors:** Laura Blanco-Hinojo, Laia Casamitjana, Jesus Pujol, Gerard Martínez-Vilavella, Susanna Esteba-Castillo, Olga Giménez-Palop, Valentín Freijo, Joan Deus, Assumpta Caixàs

**Affiliations:** 1 MRI Research Unit, Department of Radiology, Hospital del Mar, 08003 Barcelona, Spain; 21404jpn@comb.cat (J.P.); g.martinezvilavella@gmail.com (G.M.-V.); Joan.Deus@uab.cat (J.D.); 2 Centro Investigación Biomédica en Red de Salud Mental, CIBERSAM G21, 08003 Barcelona, Spain; 3 Endocrinology and Nutrition Department, Institut d’Investigació I Innovació Parc Taulí I3PT, Parc Taulí Hospital Universitari, 08208 Sabadell, Spain; lcasamitjana@tauli.cat (L.C.); olga_gimenez@yahoo.com (O.G.-P.); acaixas@tauli.cat (A.C.); 4 Medicine Department, Universitat Autònoma de Barcelona, 08193 Bellaterra, Spain; 5 Specialized Service in Mental Health and Intellectual Disability, Institut Assistència Sanitària (IAS), Parc Hospitalari Martí i Julià, 17190 Girona, Spain; susanna.esteba@ias.cat; 6 Neurodevelopment Group [Girona Biomedical Research Institute]-IDIBGI, Institute of Health Assistance (IAS), Parc Hospitalari Martí i Julià, 17190 Girona, Spain; 7 Physical Medicine and Rehabilitation Department, Parc Taulí Hospital Universitari, 08208 Sabadell, Spain; vfreijo@tauli.cat; 8 Department of Clinical and Health Psychology, Autonomous University of Barcelona, 08193 Bellaterra, Spain

**Keywords:** cerebellum, fMRI, motor system, Prader Willi syndrome

## Abstract

Severe hypotonia during infancy is a hallmark feature of Prader Willi syndrome (PWS). Despite its transient expression, moto development is delayed and deficiencies in motor coordination are present at older ages, with no clear pathophysiological mechanism yet identified. The diverse motor coordination symptoms present in adult PWS patients could be, in part, the result of a common alteration(s) in basic motor control systems. We aimed to examine the motor system in PWS using functional MRI (fMRI) during motor challenge. Twenty-three adults with PWS and 22 matched healthy subjects participated in the study. fMRI testing involved three hand motor tasks of different complexity. Additional behavioral measurements of motor function were obtained by evaluating hand grip strength, functional mobility, and balance. Whole brain activation maps were compared between groups and correlated with behavioral measurements. Performance of the motor tasks in PWS engaged the neural elements typically involved in motor processing. While our data showed no group differences in the simplest task, increasing task demands evoked significantly weaker activation in patients in the cerebellum. Significant interaction between group and correlation pattern with measures of motor function were also observed. Our study provides novel insights into the neural substrates of motor control in PWS by demonstrating reduced cerebellar activation during movement coordination.

## 1. Introduction

Prader Willi syndrome (PWS) is a rare genetic disease that is due to the loss of expression of the paternal copy of chromosome 15q11-q13 [1] and has an estimated birth incidence of 1 in 30,000 [2]. The disorder has a broad clinical expression characterized, among other features, by multiple metabolic and endocrine complications of hypothalamic origin, long lasting hyperphagia with an obsession with food leading to excessive weight gain, and mild to moderate intellectual disability associated with neurological symptoms and prominent behavioral problems [1,2,3,4,5].

In addition, alterations in the motor control in PWS entail an important part of the clinical expression that may be severe at birth. Neonatal hypotonia is a hallmark feature of PWS [1,3] characterized by a generalized reduction in muscle tone and movement accompanied by remarkable feeding difficulties. Newborns present with a poor suck that usually requires special feeding techniques [3] and results in poor weight gain and a failure to thrive during early infancy. Marked hypotonia in PWS has a transient expression and gradually improves after neonatal and infant stages [1,2,3]. Nevertheless, motor development is usually delayed and other motor symptoms appear later on [6,7], revealing an evolutive transformation of the motor control problems as the child grows up. At older ages, the PWS phenotype include a range of dyspraxia symptoms with deficient motor coordination (e.g., manual dexterity, graphomotor skills) and articulatory difficulties [3,6,8,9,10], severe postural instability, balance deficits, and gait disturbances [11,12,13], with a detectable decrease in muscle strength or weakness [10,14,15,16].

No clear pathophysiological mechanism has been yet identified accounting for the motor expression of the PWS. The extensive genetic defect in PWS is likely to interfere with brain development at multiple levels. However, the clinical characteristics of the symptoms and the evolution from a dominant hypotonia in infants to broad repercussions on the motor control in adults suggest that the motor symptoms could share, at least in part, a common discrete neural correlate rather than being a consequence of diffuse brain damage.

Current imaging studies have identified anatomical alterations in both the cerebellum and the sensorimotor cortex e.g., [8,17,18,19,20]. Functional alterations have also been reported implicating elements of the motor systems in terms of metabolism, functional connectivity at rest and abnormal activation in response to challenging stimuli related to eating behavior [5,21,22,23,24,25]. Nevertheless, to our knowledge, no studies have directly examined the potential neural mechanisms underlying the motor problems in the PWS using functional magnetic resonance imaging (fMRI) during motor challenge.

In the current study, we employed fMRI to investigate whether the brain systems involved in the motor control are dysfunctional in adults with PWS. Neural activity evoked in PWS patients during a series of motor tasks of different complexity was compared with the normal pattern identified in a reference group of healthy participants. A correlation analysis was also performed between brain activation and behavioral measures of motor performance including upper limb muscle strength, functional mobility and balance.

We hypothesized that activity in the motor system would be abnormal in the adult with PWS, and that the cerebellum could be particularly affected, as a brain structure capable of influencing each aspect of motor control. Indeed, although there are other factors that may be associated with neonatal hypotonia, such as metabolic or muscle-related peripheral factors, which could also be present in PWS, neonatal hypotonia may perhaps express to some extent a severe cerebellar dysfunction partially compensated during development, but more subtly expressed in the form of motor coordination deficits in adult PWS patients. The anatomical alterations described in the cerebellum in adults may well indicate that the cerebellum does not develop normally despite partial functional compensations.

## 2. Materials and Methods

### 2.1. Participants

The sample included 23 patients with genetically diagnosed PWS (genotype-confirmed anomaly of chromosome 15). Genetic testing showed 16 patients (70%) with interstitial deletion of the proximal long arm of chromosome 15, and 7 patients (30%) with maternal uniparental disomy or defect of the imprinting center. Patients under the age of 18 years or with non-stable medical conditions and those considered unable to follow MRI instructions were not eligible. Twenty-two healthy subjects matched by age and sex to the PWS group made up the control sample. Table 1 provides clinical characteristics of study participants. PWS patients were recruited from the Endocrinology and Nutrition Department of a Reference Center (Hospital Universitari Parc Taulí, Sabadell). Healthy subjects were hospital staff or acquaintances that participated voluntarily. A complete medical interview was carried out in healthy controls to exclude subjects with relevant medical or neurological disorders, substance abuse, psychiatric disease or undergoing medical treatment.

The study was conducted according to the guidelines of the Declaration of Helsinki. The protocol was approved by the Institutional Ethics Committee of Institut Universitari Parc Taulí of Sabadell, Barcelona (ref. END-GH-2017). All participants or their parents/guardians provided written informed consent and all PWS patients who were not able to sign, gave drawn assent.

### 2.2. Behavioral Assessment

Participants underwent selected behavioral tests of motor function in a single session prior to the MRI scanning day. The following tests were administered:

Hand grip strength was measured as an indicator of general muscle strength using the Jamar hydraulic hand dynamometer (Saehan Corporation, Changwon, Korea). Participants were instructed to sit in a chair with their feet touching the ground, shoulder adducted and neutrally rotated, with the elbow bent to 90°, the arm against the trunk, and the wrist in a neutral position [26]. Each participant’s grip strength was measured 3 times in each of 5 handle positions with each hand. Participants squeezed the dynamometer as hard as they could for 3 s. The rest period between measurements was 15 s. A practice trial at less than full force and one test trial were completed with each hand. The test took approximately 3 min to administer. The grip strength for each handle position was the maximal value of all 6 measurements. The maximal grip strength for a participant was the maximal value achieved among all 30 measurements.

Functional mobility and risk for falls was assessed by the Timed Up and Go (TUG) test [27]. Participants were instructed to sit upright with their back against an armless chair, stand up once they hear a verbal cue, walk three meters at a self-selected pace, turn around, walk back towards the chair, and sit down again in the starting position. The measure outcome was the total time (seconds) to complete the course from the verbal cue until the participant was once again fully seated in the chair. Time was measured using a manual stopwatch. The average of three trials was utilized for statistical analysis.

The Berg balance scale (BBS) [28] was used as a quantitative measure of balance. The BBS rates performance of 14 different tasks of ability to maintain positions or movements of increasing difficulty (e.g., retrieving object from floor, standing on one foot, standing with eyes closed) on a continuum from 0 (worst) to 4 (best) with a total score out of 56. A lower score indicates increased impairment of balance. It takes 20 min to complete the examination. A cut-off score of 40 is commonly used to divide patients into high risk for falls (scores < 40) and a low risk for falls (scores > 40).

### 2.3. Motor Tests during fMRI

Participants were previously given instructions concerning fMRI testing procedures and the need to remain still during acquisition. Functional MRI testing involved the performance of three manual tasks of different motor complexity [29,30]: (1) Repetitive flexion-extension of one hand. Participants were required to make repetitive self-paced opening and closing motions of the hand at a trained rate of one flexion-extension cycle every two seconds, alternating hands for 30 s each, beginning with the right hand; (2) Bimanual anti-phase repetitive flexion-extension movements. This task consisted of flexion and extension of the two hands with a phase shift of 180° between them (one hand flexes while the other extends); and (3) Repetitive sequence of fingers-to-thumb opposition movements with the right hand. Participants were instructed to consecutively connect the touch balls of the thumb with the other fingers in a complex self-paced sequence beginning with the index finger, followed by the middle finger, the ringer finger, and finally the little finger.

### 2.4. Functional MRI Acquisition

A Philips Achieva 3.0 Tesla magnet (Philips Healthcare, Best, The Netherlands), equipped with an eight-channel phased-array head coil and single-shot echo planar imaging (EPI) software (version 5.3, Philips Healthcare, Best, The Netherlands) was used for the fMRI assessment. The functional sequences consisted of gradient recalled acquisition in the steady state (time of repetition (TR), 2000 ms; time of echo (TE), 35 ms; pulse angle, 70°) within a field of view of 230 × 230 mm, with a 64 × 64-pixel matrix, and a slice thickness of 3.59 mm (inter-slice gap, 0 mm). A total of 34 interleaved slices were acquired to cover the whole brain. Three 3-min scans were acquired for each participant. Each functional time series consisted of 90 consecutive image volumes obtained during each of the 3-min assessments. The first four (additional) image volumes in each run were discarded to allow magnetization to reach equilibrium. All participants wore headphones and performed all three tasks.

For each of the three fMRI tests, we used an identical 3AB block-design paradigm consisting of six 30-s blocks, totaling 3 min in duration, which corresponded to 3 blocks of 30 s of right hand movement alternating with 3 blocks of 30 s of left hand movement in the first task, and to 3 rest (baseline) blocks of 30 s alternating with 3 movement blocks of 30 s in the second and third tasks. The examiner visually controlled task performance and gave the commands “now move” and “now pause” at the beginning and end of the respective hand movement blocks. During the rest period, participants were asked to simply lie still and not think about hand movements.

### 2.5. Functional MRI Preprocessing

Imaging data were processed using Statistical Parametric Mapping software (SPM12; https://www.fil.ion.ucl.ac.uk/spm (accessed on 18 January 2021)) implemented in Matlab (version 2016a; The MathWorks Inc, Natick, MA, USA). Preprocessing involved motion correction, spatial normalization, and smoothing by means of a gaussian kernel of full-width half-maximum 8 mm. Data were normalized to the standard SPM-EPI template and resliced to 2 mm isotropic resolution in Montreal Neurological Institute (MNI) space. All image sequences were visually inspected for potential acquisition and normalization artifacts.

#### Control of Potential Head Motion Effects

To control for the effects of head motion, we adopted the following approach: (i) time series were aligned to the first functional image (of each time series) in each participant using a least squares minimization and a 6-parameter (rigid body) spatial transformation. (ii) We included 6 motion-related regressors in the first-level (single-subject) analyses. (iii) Within-subject, censoring-based MRI signal artifact removal (scrubbing) [31] was used to discard motion-affected volumes. For each participant, mean inter frame motion measurements [32] served as an index of data quality to flag volumes of suspect quality across the run. At points with mean inter frame motion >0.3 mm, we discarded the corresponding volume and the succeeding volume. Using this procedure, a mean (±SD; range) of 0.4 (±1.0; 0–4) volumes in the control group and 7.6 (±9.5; 0–30) in the PWS group were removed for the first task analysis. For the analysis of the second task, 0.6 (±1.1; 0–3) volumes in the control group and 6.4 (±7.5; 0–24) in the PWS group were removed. Volume removal for the third task was 0.5 (±1.4; 0–6) volumes in controls and 5.0 (±5.3; 0–17) in PWS. (iv) A minimum of 120 s (60 volumes) with no motion artifacts after scrubbing was required for participants to be included in the analyses. As a result of this criteria, from the initial sample of 23 subjects in the PWS group, data from one patient was removed in the simple task analysis, and data from another patient was removed in the analysis of the bimanual task. (v) The remaining potential motion effects were controlled by including a motion summary measurement for each participant as a covariate in the group analyses in SPM [32].

### 2.6. Statistical Analysis

#### 2.6.1. Behavioral Data

Student’s *t* test was used to compare mean differences between the PWS and the control group in terms of demographic characteristics and behavioral ratings. Pearson’s chi-squared test was used to assess the relationships between categorial variables. Visual analyses and Shapiro-Wilk tests showed non-normal distributions in the scores of the TUG test and the BBS. Thus, the non-parametric Mann-Whitney test was used to study potential group differences in these variables. The statistical analyses on the behavioral data were conducted with the Statistical Package for the Social Sciences (SPSS v19 (version 19, SPSS Inc., Chicago, IL, USA)).

#### 2.6.2. Functional MRI Data

To obtain individual maps of brain activity evoked during motor challenge, a boxcar regressor was generated considering the three blocks of the baseline condition and the three blocks of the test condition, and applying a hemodynamic delay of 4 s. Contrast “right hand < left hand” and “right hand > left hand” images were estimated for each participant in the unimanual flexion-extension task. The contrasts “baseline < motion” (activation) and “baseline > motion” (deactivation) were estimated instead for both the bimanual anti-phase flexion-extension and the finger-to-thumb opposition tasks. Resulting first-level SPM contrast images for each subject were carried forward to group-level random-effects analyses. One-sample *t* test designs were used to generate group activation maps. Two-sample *t* tests were used to compare brain activation between the PWS and the control group. In addition, whole-brain correlation analyses were performed to map the possible relationships between brain activation and maximal grip strength, time to complete the TUG test, and BBS scores separately and for each task.

Thresholding criteria. In whole-brain analyses, clusters > 2.3 mL (290 voxels) at a height threshold of *p* < 0.005 were considered, which satisfied the family-wise error (FWE) rate correction of *p*_FWE_ < 0.05, according to Monte-Carlo simulations.

## 3. Results

### 3.1. Behavioral Data

Descriptive statistics for all the measures are presented in Table 1. Maximal (±SD, range) hand grip strength was 17.8 (±5.7, 10–34) kg for patients, and 34.3 (±10.5, 20–58) kg for controls (t = −6.4, *p* < 0.001). The mean grip strength for each handle position for each group is shown in Table 1. In both groups, maximal grip strength was achieved at position 2, which indicates that patients were cooperative during the test [33]. There was a significant difference in the average time employed in the TUG test between PWS patients and controls (U = 480.0, *p* < 0.001), and PWS patients’ score in the BBS test was also significantly lower than that for controls (U = 28.5, *p* < 0.001). In the PWS group, there was a significant negative association between the scores in the TUG and BBS tests (rs = −0.61, *p* = 0.02) such that less time in the TUG test was associated with higher scores in the BBS. This association was not observed in the control group, probably due to a ceiling effect of the scores in the BBS test.

### 3.2. Functional MRI Data

**Figure 1:**
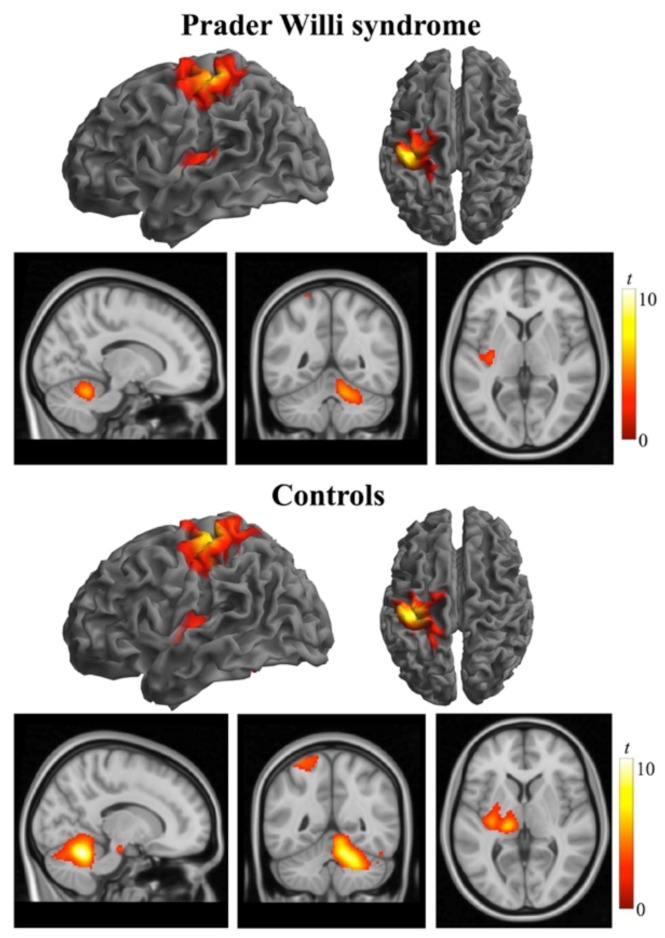
Brain activation (one sample *t*-tests) obtained during the flexion-extension task with the right hand in individuals with Prader Willi syndrome (top rows) and control subjects (bottom rows). For each group, the functional data are displayed on the lateral and dorsal cortical surfaces (white background) and superimposed on a high-resolution anatomical template (sagittal, coronal, and axial views, respectively; black background) using SPM. Activations are thresholded at *p*_FWE_-corrected < 0.05. Color bars represent *t* values. Right in axial and coronal views corresponds to the right hemisphere.

The “across-hand” analysis of the first task consisted of the comparison of the right-versus-left hand activation. In both groups, repetitive opening and closing of the hand caused a localized significant increase in MR signal intensity in the contralateral Rolandic region that was strictly located in the primary motor and somatosensory cortices, anterior and posterior to the central sulcus respectively, with a peak maximum at the cortical representation of the hand, as well as in the contralateral putamen extending to the posterior insula, and cerebellum ipsilateral to the hand that was performing the task (Figure 1, Table S1). No significant between-group differences were found for this task.

**Figure 2:**
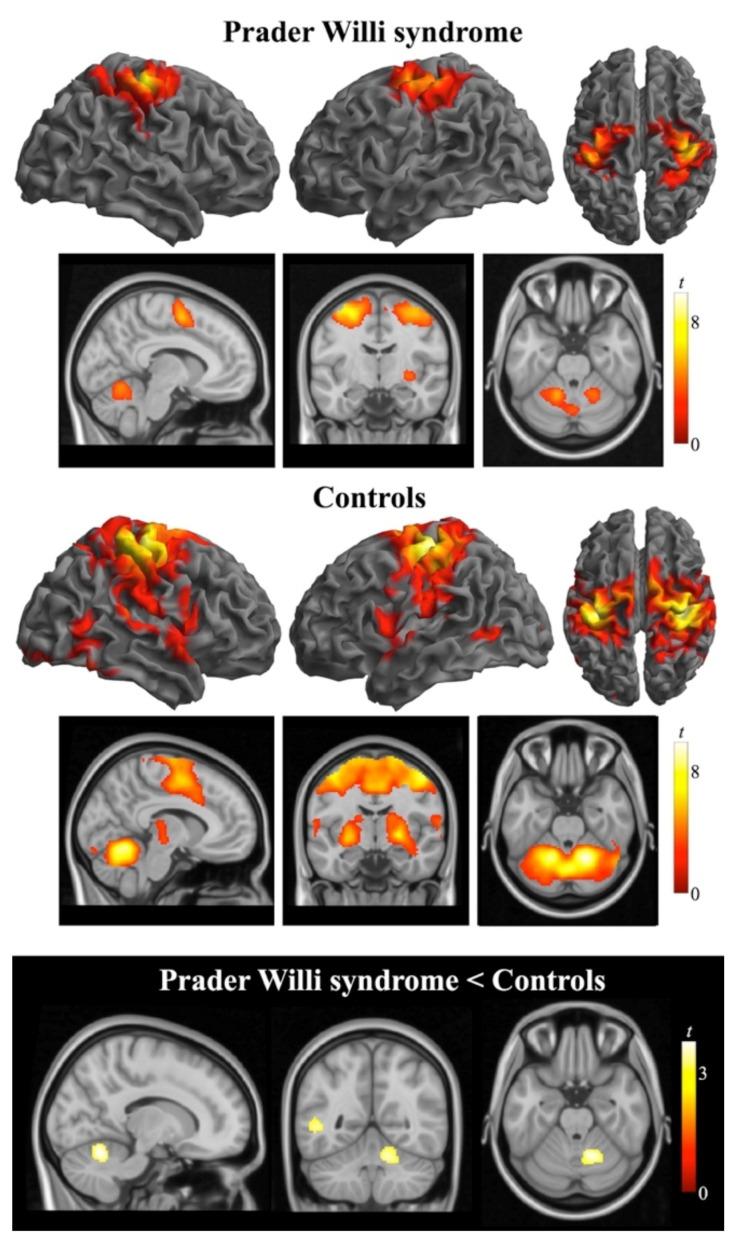
Brain activation (one sample *t*-tests) obtained during the bimanual anti-phase flexion-extension task in individuals with Prader Willi syndrome (top rows) and control subjects (mid rows). The brain views in the bottom row illustrate between-group differences in whole brain activation (two sample *t*-test). Activations are thresholded at *p*_FWE_-corrected < 0.05. Color bars represent *t* values. Right in axial and coronal views corresponds to the right hemisphere.

The analysis of the second task consisted of the comparison of brain activation during bimanual anti-phase flexion-extension movements with the resting condition. As opposed to the analysis of the first task, this analysis did not produce cancellation of shared (non-localized) activity and, therefore, it allowed further functional identification of non-primary cortex activations. The control group showed activation in the sensorimotor strip, premotor and supplementary motor areas, superior parietal cortex, central operculum, thalamus, putamen and pallidum in the basal ganglia, and cerebellum bilaterally. The pattern of activation in the PWS group was qualitatively similar, albeit less extensive particularly in subcortical regions, and included bilateral changes occurring in sensorimotor, premotor and supplementary motor cortices, and cerebellum (Table S2). Figure 2 illustrates the motor-related activation shown by both groups. For the reverse contrast, that is, resting > movement, a significant deactivation was found in the PWS group in left temporal and occipital cortices that was not observed in the control group. Significant between-group differences were identified in the right cerebellar cortex involving lobules IV/V and VI surrounding the primary fissure, and in a small cluster in the left temporo-occipital cortex.

**Figure 3:**
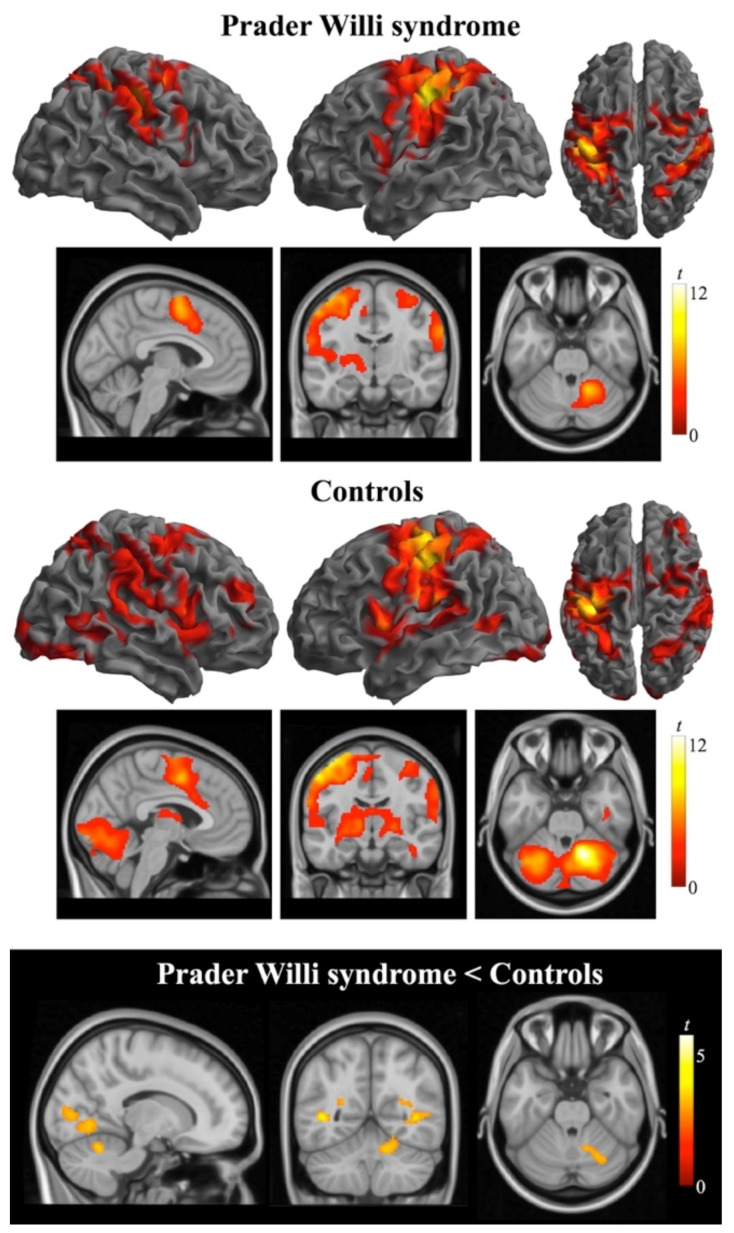
Brain activation obtained during the fingers-to-thumb opposition task in individuals with Prader Willi syndrome (top rows) and control subjects (mid rows). The brain views in the bottom row illustrate between-group differences in whole brain activation. Activations are thresholded at *p*_FWE_-corrected < 0.05. Color bars represent *t* values. Right in axial and coronal views corresponds to the right hemisphere.

In the finger-opposition task, neural activation was conspicuous in both groups in the contralateral (left) sensorimotor hand area and ipsilateral cerebellum, thus resembling the activation pattern observed in the simpler unimanual task, but similarly to the bimanual task, such a fine finger movements also recruited ipsilateral and contralateral areas surrounding the primary sensorimotor cortex, thalamus, basal ganglia extending to the insula and opercula bilaterally, and contralateral cerebellum. In the control group, activation was also found in ipsilateral and, to a lesser extent, contralateral prefrontal cortex and temporo-occipital cortex bilaterally. In the PWS group, the maximal activation was found in the posterior bank of the central sulcus in the region that contains the sensorimotor representation of the hand; however, subcortical activation was restricted to the contralateral thalamus, pallidum and putamen, and ipsilateral cerebellum (Figure 3, Table S3). For the reverse contrast (resting > movement), the PWS group demonstrated deactivation in widespread regions in the occipital lobe. Stronger activation in the control group compared with the PWS group was found in the cerebellar cortex (lobules V/VI) ipsilateral to the hand movement. Conversely, patients did not demonstrate increased activation than controls in any region.

In order to study the effect of the genetic subtype, PWS patients were split in 2 groups: DEL (*n* = 16) and UPD (*n* = 7). Groups did not differ in age, sex or IQ. No significant differences in brain activation were observed between the two groups.

**Figure 4:**
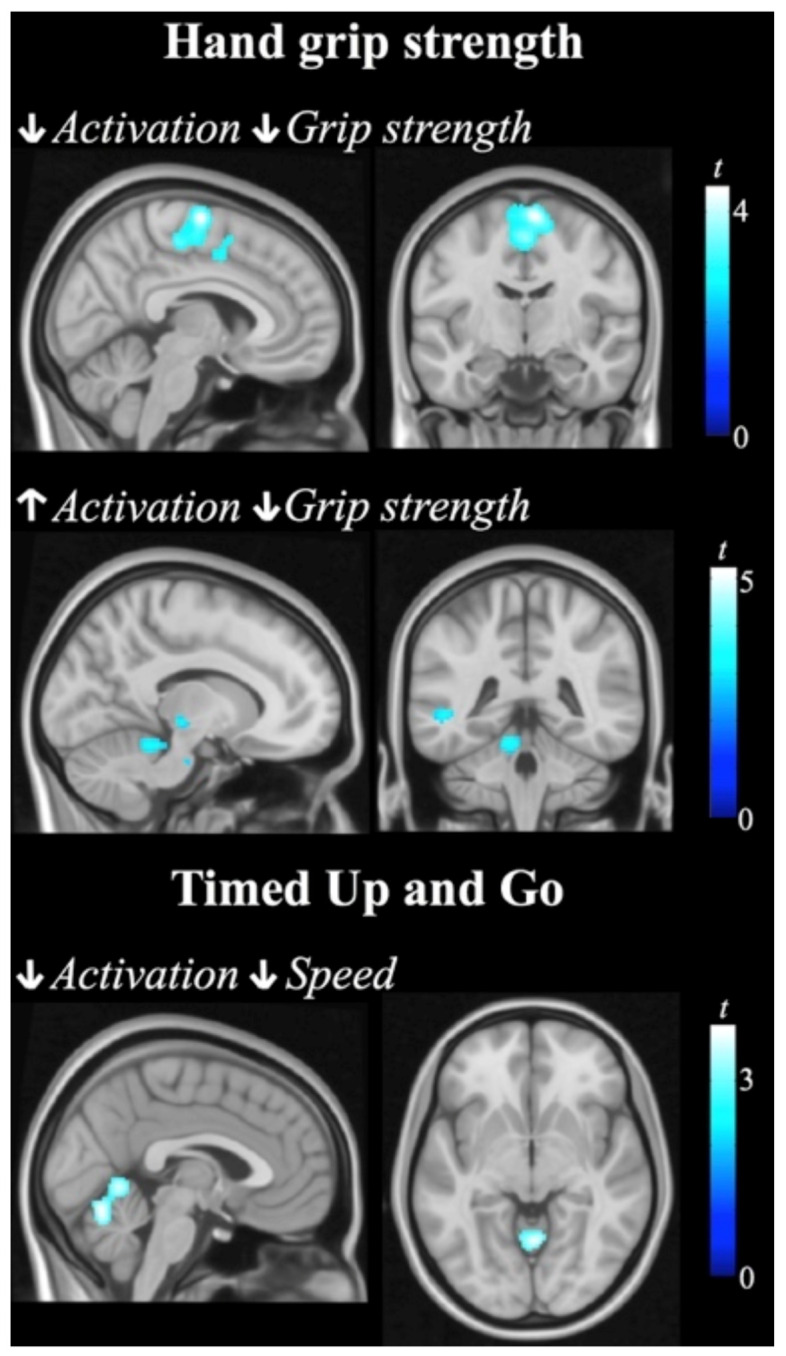
Correlation between brain activation and behavior scores. Significant interactions between group and correlation pattern are reported. Individuals with Prader Willi syndrome showed a stronger (than controls) positive association between activation in the supplementary motor area and hand grip strength (top row), stronger negative association between activation in the cerebellum and hand grip strength (mid row), and weaker association between activation in the cerebellar vermis and score in the Timed Up and Go test (bottom row). Color bars represent *t* values. Right in coronal views corresponds to the right hemisphere.

In the analysis of correlations between behavioral measurements and brain activation, significant interactions between group and correlation pattern were found involving brain structures related to motor control. Specifically, during performance of the bimanual anti-phase task, PWS patient versus controls showed stronger positive correlation between activation in a cluster encompassing the paracentral lobule and supplementary motor cortex and maximal hand grip strength. That is, grip strength decreases as SMA activation decreases in PWS, whilst in the control group the direction of the association was the opposite. Less activation in the cerebellar vermis was associated with better performance in the TUG test in the control group, whereas the association was the reverse but not significant in the PWS group. Similarly, PWS patients showed weaker (than controls) positive correlation between left premotor cortex activation and BBS scores. Finally, PWS showed stronger (than controls) negative correlation between hand grip strength and activation in the left thalamus and cerebellum during the fingers-opposition task. Brain areas where the magnitude of activation during the fMRI tasks was associated with changes in behavioral measurements are summarized in Table S4 and illustrated in Figure 4 and Figure S1.

## 4. Discussion

In the present study, we aimed to investigate the brain’s motor system in adult PWS patients using fMRI with motor activation paradigms of different complexity. Selected behavioral testing additionally served to assess general aspects of motor function (i.e., muscle strength, mobility, and balance). We found that patients displayed altered motor performance and reduced brain activation during motor tasks compared with healthy controls. In line with our hypothesis, statistically significant between-group differences involved the cerebellum. Significant interactions between group and correlation pattern with measures of motor function were also observed.

In the simple task, consisting in the repetitive flexion-extension of one hand, control subjects demonstrated the expected finding of robust activations in primary sensorimotor cortex and thalamus contralateral with respect to the hand participating in the movement, and ipsilateral cerebellum. With increasing task complexity, that is, repetitive bimanual anti-phase flexion-extension movements and fingers-to-thumb opposition movements, a greater number of secondary regions and bilateral subcortical areas were additionally recruited, which is in accordance with the results of previous fMRI and positron emission tomography (PET) studies using equivalent tasks e.g., [29,30,34,35,36]. In addition to task complexity, movement frequency could be a relevant factor in determining the pattern of brain activation. We used a trained self-paced rate of two seconds (0.5 Hz) for the performance of the motor tasks. Increased task demands due to increased movement speed may result in different activation effects particularly in the cerebellum and the basal ganglia, although frequency variations in the 0.2–2 Hz range appear not to be a relevant source of the variability of the fMRI results in healthy subjects [37,38]. Overall, the pattern of brain activation in the PWS group was similar, albeit less extensive, than in the control group and included the cortical and subcortical regions typically involved in motor processing. However, while our data showed no group differences in the simplest task, increasing task demand resulted in significantly lesser activation in patients in the right cerebellar hemisphere, comprising lobule V in the anterior cerebellum extending into lobule VI, coinciding with the hand representation of the anterior cerebellar homunculus [39]. Brain motor-related activation abnormalities may thus be not apparent in PWS during relatively simple manual tasks, but they are evidenced instead when the tasks are more demanding implicating fine finger movements in a sequence or require the coordinated involvement of both hands, which is consistent with the manual dexterity and finger coordination difficulties that characterize the disorder [7,10].

The cerebellum has an important role in the control of fine voluntary movements, motor coordination and motor timing [40,41,42]. Reciprocal pathways interconnect the cerebellum via the pontine nuclei (afferents) and the thalamus (efferents) with cerebral components of the motor system following a topographical distribution [39,43,44], with lobules I–V in the anterior lobe, and lobules VI and VIII from the posterior lobe being mainly sensorimotor [45]. The cerebellum is actively involved in the integration of sensory and motor events, receiving extensive sensory input and modifying the descending motor pathways to bring movements accurately to its desired endpoint [41,43,46], and is essential for the production of smooth, continuous movements [40] and the automation of motor behaviors [42]. Decreased cerebellar activation in our study may reflect a deficit in automatization of the motor tasks [47], with greater dependence on cortical motor regions associated with effortful movement and reduced efficiency in motor execution. Cerebellar activation is strongly corelated with movement frequency and quantity [42] and is associated with advanced performance in finger-tapping tasks [48].

PWS patients typically score below the normal range on standardized motor performance tests [7]. Consistent with the literature [10,15], our data showed that participants with PWS exhibited significantly lower maximal muscle force in upper limbs, greater average time in the execution of the walking task, as well as poorer balance compared to control participants. Nevertheless, only one patient exhibited score < 40 in the balance scale indicating high risk for falls, and all except the same patient took fewer than 20 s in the TUG test, which indicates that the PWS participants in our study are mostly independent for basic moves [27]. In addition to the group differences, we found several group interactions between clinical measurements and brain activation implicating the cerebellum and other motor regions. Remarkably, there was a significant interaction in the correlation between functional mobility and brain activation in the cerebellar vermis, a medial zone in the cerebellum that is particularly concerned with whole-body posture and locomotion [49]. This interaction was driven by a significant positive association in the control group (i.e., better performance, greater activation in this cerebellar region during the bimanual task) that was not demonstrated by the PWS group. The presence of a correlation between walking ability and cerebellar activation in healthy subjects and the lack of such relationship in PWS patients suggest that, beyond abnormal body composition and obesity [12], the cerebellum plays a role in gait difficulties in this population.

Neuroanatomical and imaging studies have described cerebellar abnormalities [19,50,51] and reduced cerebellar volume in both children and adults with PWS compared with control samples [17,20,52]. Using voxel-based morphometry, findings from our group [8] and others [53] have revealed decreased relative white matter volume in the cerebellum. It is possible that our finding of abnormal cerebellar activation in PWS participants results directly from tissue volume reduction in this region. However, deficient connections in the context of cerebrocerebellar sensorimotor circuits are also plausible. Indeed, transcranial magnetic stimulation changes in PWS suggest abnormal (hypo) excitability of the motor cortical areas [54], which is modulated by cerebellar input [44,55], and functional connectivity alterations between major components of the motor system have been reported in PWS [5,25]. Further studies examining the integrity of the connections between the cerebellum and the other elements in cerebrocerebellar sensorimotor circuits are of interest to determine the extent of the cerebellar dysfunction in PWS.

Accurate control of head motion effects may be a strength of our studies; however, fMRI activation findings can be complicated by the level of intellectual disability present in PWS individuals which may interfere with task complexity and represent a potential confound related to cognitive demand. Nevertheless, we selected only relatively complex tasks, and subjects were trained on and they practiced the tasks until the authors verified that all participants performed the tasks correctly so that they were considered well learned before the fMRI scan, which does not support that PWS’ cerebellar dysfunction is simply the result of their IQ. Future studies using a control group of subjects with a similar intellectual disabilities may help elucidating this issue. The study is also limited by its cross-sectional nature with a relatively small sample size, which might prevent us from drawing general conclusions.

In summary, our study provides novel insights into the neural substrates of motor control in PWS by demonstrating reduced cerebellar activation during movement coordination. Prominent hypotonia in infants and subsequent motor problems that continue into childhood are compatible with a poor cerebellar function as a key pathophysiological factor. Our findings reinforce the role of the cerebellar dysfunction in PWS and indicate that it may be also expressed in adulthood. Investigation of the neural mechanisms underlying motor development in PWS may help improving the effectiveness of current therapies and eventually designing new interventions for the motor difficulties that characterize these patients.

## Figures and Tables

**Table 1:** Demographic and clinical characteristics of study participants.

	Prader Willi (*n* = 23)	Control (*n* = 22)
Age, year	30.6 ± 10.1 (18–53)	29.8 ± 9.2 (19–46)
Sex, M/F	11/12	10/12
Body mass index, kg/m^2^	34.9 ± 7.4 (23.3–53.2)	22.0 ± 1.8 (18.7–25.2)
Laterality (R/L)	16/7	17/4 *
IQ K-BIT—Total score	70.3 ± 14.3 (48–99) ^#^	
Genetic diagnosis, no.		
Type I deletion	8	
Type II deletion	6	
Atypical deletion	2	
Uniparental disomy	4	
Imprinting defect	3	
Motor function		
Hand grip strength, max. kg		
Handle position 1	13.7 ± 5.8 (6–32)	28.2 ± 8.8 (17–54) *
Handle position 2	17.4 ± 5.8 (10–34)	33.8 ± 10.6 (20–58) *
Handle position 3	16.3 ± 5.4 (9–33)	31.2 ± 9.7 (19–52) *
Handle position 4	14.2 ± 5.3 (6–30)	27.3 ± 8.8 (15–43) *
Handle position 5	12.0 ± 4.7 (6–23)	22.6 ± 8.5 (12–40) *
Timed Up and Go, sec	10.5 ± 6.6 (6.3–38.4)	5.2 ± 0.6 (3.9–6.5) *
Berg Balance Scale (range 0–56)	51.5 ± 6.3 (25–56)	55.7 ± 0.5 (55–56) *

Values are expressed as group: mean ± standard deviation (range). IQ-KBIT, Intelligence Quotient—Kaufman Brief Intelligence Test. ^#^ *n* = 18. * *n* = 21 (one control subject did not undergo behavioral testing).

## Data Availability

The data that support the findings of this study are available from the corresponding author upon reasonable request.

## Supplementary Materials

The following are available online at https://www.mdpi.com/article/10.3390/jcm10153320/s1, Figure S1: plots of the correlations between activation and behavioral measurements, Table S1: fMRI brain activation during the unimanual flexion-extension task, Table S2: fMRI brain activation and deactivation during the bimanual anti-phase flexion-extension task, Table S3: fMRI brain activation and deactivation during the finger-to-thumb opposition task, Table S4: correlations between activation measurements and behavior scores.
