# Supplementary material for: Cerebellar Dysfunction in Adults with Prader Willi Syndrome
Source: J Clin Med. 2021 Jul 28;10(15):3320. doi: 10.3390/jcm10153320 (PMC8347444; doi:10.3390/jcm10153320)
Supplement: Supplementary file 1 [file jcm-10-03320-s001.zip › jcm-1279629-supplementary.pdf]

Table S1. fMRI brain activation during the unimanual flexion-extension task.

	Control Subjects		PWS Patients	
	MNI x y z	T	MNI x y z	T
Right hand				
R Cerebellum	12 -54 -22	10.7	14 -52 -24	5.7
L Thalamus	-16 -22 2	6.8	-	-
L Putamen	-32 -18 2	5.4	-32 -8 -8	4.3
L Sensorimotor Cortex	-38 -26 50	9.5	-40 -28 58	8.9
Left hand				
L Cerebellum	-12 -56 -18	10.0	-12 -56 -16	7.5
R Sensorimotor Cortex	26 -16 62	9.2	38 -22 60	8.9
R Supplementary Motor Area	8 -6 48	5.2	8 -8 56	4.3
R Putamen/Insula	38 -22 16	4.2	48 -16 12	5.9

x, y, z are coordinates (mm) given in Montreal Neurological Institute (MNI) space. Statistics at corrected threshold P_{FWE}<0.05 using Monte Carlo simulations. L, left. R, right.

Table S2. fMRI brain activation and deactivation during the bimanual anti-phase flexion-extension task.

	Control Subjects		PWS Patients	
	MNI x y z	T	MNI x y z	T
Task > Rest				
L Sensorimotor Cortex	-38 -26 56	10.3	-40 -26 58	6.5
R Sensorimotor Cortex	36 -24 50	9.4	40 -22 58	8.2
L Premotor Cortex	-28 -18 72	8.5	-26 -12 58	6.8
R Premotor Cortex	24 -14 72	6.8	34 -6 62	4.5
L Supplementary Motor Area	-4 -4 52	6.6	-12 -2 56	5.8
R Supplementary Motor Area	8 -2 48	6.0	12 -2 58	6.0
R Cerebellum	18 -54 -26	9.7	22 -52 -30	4.3
L Cerebellum	-6 -60 -14	8.8	-16 -52 -26	4.9
R Thalamus	16 -18 2	5.5	-	-
L Thalamus	-16 -20 4	4.5	-	-
R Basal Ganglia	24 -8 0	7.1	30 -12 -4	3.9
L Basal Ganglia	-28 -8 -4	5.7	-	-
L Temporo-Occipital Cortex	-46 -62 2	4.6	-	-
Task < Rest				
L Occipital Cortex	-	-	-32 -96 10	4.3
L Temporal Cortex	-	-	-48 -44 2	4.2

	PWS Patients < Control Subjects		
	Cluster size, ml	MNI x y z	T
R Cerebellum	3.1	14 -58 -22	3.9
L Temporo-Occipital Cortex	2.6	-46 -62 0	3.6

x, y, z are coordinates (mm) given in Montreal Neurological Institute (MNI) space. Statistics at corrected threshold P_{FWE}<0.05 using Monte Carlo simulations. L, left. R, right.

Table S3. fMRI brain activation and deactivation during the finger-to-thumb opposition task.

	Control Subjects		PWS Patients	
	MNI x y z	T	MNI x y z	T
Task > Rest				
L Sensorimotor Cortex	-38 -26 52	11.0	-50 -24 44	9.9
L Supplementary Motor Area	-4 2 48	7.3	-6 0 56	7.0
R Supplementary Motor Area	8 4 50	5.7	2 0 64	6.5
L Premotor Cortex	-40 -16 62	8.7	-32 -10 60	7.3
R Premotor Cortex	32 2 60	5.3	32 -2 56	5.8
L Superior Parietal Cortex	-40 -42 56	6.8	-30 -46 62	8.2
R Superior Parietal Cortex	36 -56 62	4.5	38 -36 46	8.6
R Cerebellum	16 -54 -26	12.9	18 -52 -30	7.3
L Cerebellum	-32 -64 -32	7.7	-	-
L Thalamus	-14 -20 2	7.7	-14 -20 0	5.0
R Basal Ganglia	22 -6 4	5.7	-	
L Basal Ganglia	-20 0 4	6.7	-20 2 2	5.6
L Posterior Temporal Cortex	-52 -38 16	7.3	-	-
R Anterior Prefrontal Cortex	34 42 22	4.7	-	-
L Anterior Prefrontal Cortex	-28 52 24	3.8	-	-
Task < Rest				
R Occipital Cortex	-	-	24 -64 22	4.9
L Precuneus	-	-	-24 -62 16	4.7

	PWS Patients < Control Subjects		
	Cluster size, ml	MNI x y z	T
L Posterior Temporal Cortex	2.5	-50 -40 14	4.3
R Occipital Cortex	31.2	38 -78 -12	5.8
R Cerebellum	*	28 -70 -18	4.0

x, y, z are coordinates (mm) given in Montreal Neurological Institute (MNI) space. Statistics at corrected threshold P_{FWE}<0.05 using Monte Carlo simulations. L, left. R, right. *, same cluster.

Table S4. Correlations between activation measurements and behavior scores (group by correlation pattern interactions).

	Cluster size, ml	MNI x y z	T
Hand grip strength**			
PWS pos. corr. > Control pos. corr.			
Paracentral lobule/SMA	8.9	6 -10 68	4.4
Hand grip strength***			
PWS neg. corr > Control neg. corr			
Cerebellum	6.1	-4 -20 -24	5.2
Thalamus	3.9	-20 -26 2	4.4
Timed up-and-go test**			
PWS pos. corr < Control pos. corr			
Cerebellum	2.8	2 -58 -4	3.8
Berg Balance Scale*			
PWS neg. corr. < Control neg. corr			
Somatosensory Cortex/ Supramarginal gyrus	5.1	64 -44 38	5.0
Supramarginal gyrus	3.1	-50 -44 58	3.8
Berg Balance Scale**			
PWS pos. corr. < Control pos. corr			
Premotor Cortex	2.6	-24 6 50	4.4

*Unimanual flexion-extension task. **Bimanual anti-phase flexion-extension task. ***Fingers-to-thumb opposition task. x, y, z are coordinates (mm) given in Montreal Neurological Institute (MNI) space. SMA, Supplementary motor area. Statistics correspond to a corrected threshold $P_{FWE}<0.05$.

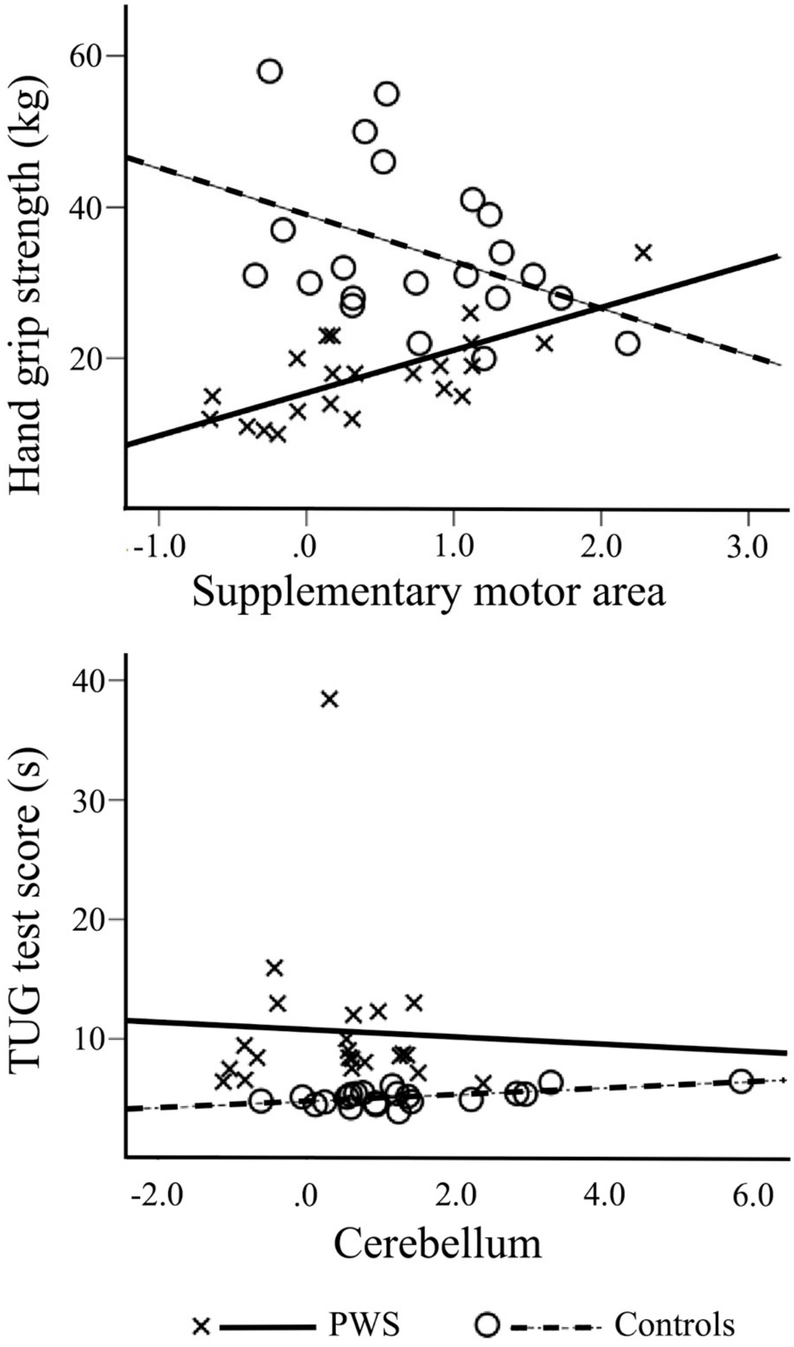

Figure S1. Plots of the correlations between activation and behavioral measurements. Top plot, individuals with Prader Willi syndrome (crosses, bold line) showed stronger positive correlation between activation in the supplementary motor area during the bimanual anti-phase flexion-extension task and maximal hand grip strength, as assessed with the JAMAR dynamometer, compared with controls (circles, dashed line) that showed a negative correlation (interaction t= 4.4, p= 0.0004 at x, y, z: 6 -10 68, displayed on top Figure 4). Bottom plot, individuals with Prader Willi syndrome showed weaker negative correlation between activation in the cerebellum during the bimanual task and total time employed in the TUG test compared with controls that showed a positive correlation (interaction t= 2.8, p= 0.0002 at x, y, z: 2 -58 -4, displayed on bottom Figure 4). TUG, Timed Up-and Go. PWS, Prader Willi syndrome.
